# Prediction of Methionine and Homocysteine levels in Zucker diabetic fatty (ZDF) rats as a T2DM animal model after consumption of a Methionine-rich diet

**DOI:** 10.1186/s12986-018-0247-1

**Published:** 2018-02-10

**Authors:** Nayoung Han, Jung-woo Chae, Jihyun Jeon, Jaeyeon Lee, Hyun-moon Back, Byungjeong Song, Kwang-il Kwon, Sang Kyum Kim, Hwi-yeol Yun

**Affiliations:** 1 College of Pharmacy and Research Institute of Pharmaceutical Sciences, Seoul National University, 1 Gwanak-ro, Gwanak-gu, Seoul 08826 Republic of Korea; 2 College of Pharmacy, Chungnam National University, 99 Daehak-ro, Yuseong-gu, Daejeon 34134 Republic of Korea; 3 New Drug Development Center, Osong Medical Innovation Foundation, 123 Osongsaengmyeong-ro, Osong-eup, Heungdeok-gu, Cheongju-si, Chungbuk 28160 Republic of Korea; 4 Drug Discovery Center, JW Pharmaceutical, 2477 Nambusunhwan-ro, Seocho-gu, Seoul 06725 Republic of Korea

**Keywords:** Methionine metabolism cycle, Homocysteine, Mathematical model, Methionine-rich diet, Type 2 diabetes mellitus (T2DM)

## Abstract

**Background:**

Although alterations in the methionine metabolism cycle (MMC) have been associated with vascular complications of diabetes, there have not been consistent results about the levels of methionine and homocysteine in type 2 diabetes mellitus (T2DM). The aim of the current study was to predict changes in plasma methionine and homocysteine concentrations after simulated consumption of methionine-rich foods, following the development of a mathematical model for MMC in Zucker Diabetic Fatty (ZDF) rats, as a representative T2DM animal model.

**Method:**

The model building and simulation were performed using NONMEM® (ver. 7.3.0) assisted by Perl-Speaks-NONMEM (PsN, ver. 4.3.0). Model parameters were derived using first-order conditional estimation method with interactions permitted among the parameters (FOCE-INTER). NCA was conducted using Phoenix (ver. 6.4.0). For all tests, we considered a *P*-value < 0.05 to reflect statistical significance.

**Results:**

Our model featured seven compartments that considered all parts of the cycle by applying non-linear mixed effects model. Conversion of S-adenosyl-L-homocysteine (SAH) to homocysteine increased and the metabolism of homocysteine was reduced under diabetic conditions, and consequently homocysteine accumulated in the elimination phase.

Using our model, we performed simulations to compare the changes in plasma methionine and homocysteine concentrations between ZDF and normal rats, by multiple administrations of the methionine-rich diet of 1 mmol/kg, daily for 60 days. The levels of methionine and homocysteine were elevated approximately two- and three-fold, respectively, in ZDF rats, while there were no changes observed in the normal control rats.

**Conclusion:**

These results can be interpreted to mean that both methionine and homocysteine will accumulate in patients with T2DM, who regularly consume high-methionine foods.

## Background

The methionine metabolism cycle (MMC) plays important roles in regulating both methionine and cysteine homeostasis [1], and is related with functions in terms of cell growth and development under normal conditions [2, 3]. As a universal key intermediate in the MMC, homocysteine is not obtained from the diet, but is remethylated to methionine, or converted to cysteine by the trans-sulfuration pathway. It is well-known that elevated homocysteine levels are associated with the early development of cardiovascular diseases [4], especially increasing the risk in patients with diabetes mellitus (DM) [5, 6]. A study performed in type 1 DM (T1DM) patients revealed an association between homocysteine levels and diabetic complications [7], and recent systematic review with a meta-analysis studies concluded that high levels of homocysteine may be associated with type 2 DM (T2DM) progression [8] and the development of vascular complications of diabetes [9]. This is because high blood glucose status, caused by insulin-resistance conditions like T2DM, induces decrements of methionine transmethylation, homocysteine transsulfuration, and clearance in type 2 diabetic subjects [10], leading to increased concentrations of plasma homocysteine [5, 8]. On the other hand, some studies reported that the levels of homocysteine were either reduced or normal under diabetic conditions [11, 12]. These inconsistent results seem to be attributable to varying extents of hepatic and renal dysfunction [11, 13], the methionine content of the diet [14, 15], and the clinical status of diabetic disease [12, 16, 17]. Here, we focused on the fact that a high-fat diet affects the metabolism of both methionine and homocysteine in a diabetic rat model [14]. Thus, 0we hypothesized that long-term administration of a high-methionine diet to T2DM rats with normal renal function may create metabolic changes, culminating in an elevated level of circulating homocysteine. To verify this hypothesis, mathematical modeling of methionine metabolism was required to predict the levels of homocysteine derived from given amounts of methionine.

Although some earlier studies had developed several mechanistic mathematical modeling approaches in an effort to understand the MMC, these models specially focused on and explained the systematic changes in cellular metabolism of methionine and homocysteine [18–20], making it difficult to apply the theoretical predictions of such models to real in vivo situations. Therefore, the objective of the current study was to develop a mathematical model of the MMC using in vivo data from T2DM rats. And we aimed to predict plasma methionine and homocysteine concentrations using the model, following the ingestion of a methionine-rich diet.

## Methods

### Study design

A single dose of 0.8 mmol/kg methionine (a mixture of L-methionine 0.6 mmol/kg and L-methionine-d_4_ 0.2 mmol/kg) was given intravenously to both the Zucker diabetic fatty (ZDF) rats (ZDF/Gmi *fa*/*fa*) [21] and the controls (ZDF/Gmi *fa*/?). Blood samples were collected at 0, 10, 30, 60, 120, 210, 300, and 420 min after administration. Methionine and homocysteine concentrations were determined using LC-ESI/MS/MS; the details of the study design were described earlier [14].

### Mechanistic modeling of the methionine metabolism cycle (MMC)

**Fig. 1:**
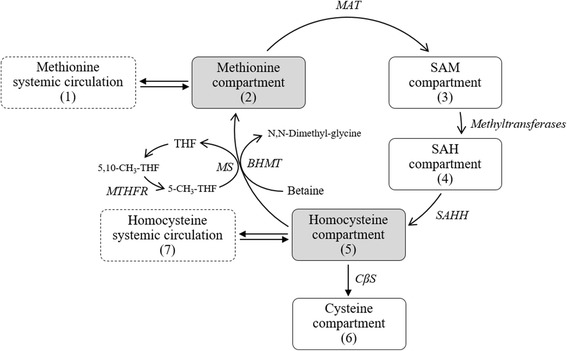
Schematic illustrations of Mechanistic Methionine Cycle (MMC) model

All parameters of the MMC model were transformed to in vivo scales, with consideration of physiological aspects, including liver volume and systemic circulation of methionine. The rate constants of distribution between compartments were simplified, by reference to prior data, suggesting that biological processes followed generally first-order kinetics. The MMC model featured a total of seven compartments associated with the five compartments of methionine, homocysteine, *S*-adenosylmethionine (SAM), *S*-adenosyl- L-homocysteine (SAH), cystathione, and the other two compartments addressing systemic distribution of methionine and homocysteine (Fig. 1). The movements of each component among compartments were described by the following differential equations (Eq):

1 $$ dA(1)/ dt=-{K}_{CM}\cdot A(1)+{K}_{MC}\cdot A(2) $$

2 $$ dA(2)/ dt=-{K}_{MS}\cdot A(2)+{K}_{HM}\cdot A(5)-{K}_{EL}\cdot A(2)-{K}_{MC}\cdot A(2)+{K}_{CM}\cdot A(1) $$

3 $$ dA(3)/ dt={K}_{MS}\cdot A(2)-{K}_{SS}\cdot A(3) $$

4 $$ dA(4)/ dt={K}_{SS}\cdot A(3)-{K}_{SH}\cdot A(4) $$

5 $$ dA(5)/ dt={K}_{SH}\cdot A(4)-{K}_{HM}\cdot A(5)-{K}_{HC}\cdot A(5)-{K}_{HP}\cdot A(5)+{K}_{PH}\cdot A(7) $$

6 $$ dA(6)/ dt={K}_{HC}\cdot A(4) $$

7 $$ dA(7)/ dt=-{K}_{PH}\cdot A(7)+{K}_{HP}\cdot A(5) $$

*K*_*CM*_ was the rate constant for the movement of methionine from compartment 1 to 2, *K*_*MC*_ was for movement from compartment 2 to 1, and *K*_*MS*_ from compartment 2 to 3 (correspondence of methionine adenosyl transferase [MAT]). *K*_*EL*_ represented the rate of methionine elimination, *K*_*SH*_ was the transformation rate constant from SAH to homocysteine (correspondence of SAH hydrolase [SAHH]), *K*_*HM*_ was the transformation rate constant from homocysteine to methionine (corresponding to the activity of betaine-homocysteine methyltransferase [BHMT]), and *K*_*HC*_ was the transformation rate constant from homocysteine to cysteine (corresponding to the activity of cystathionine beta-synthase [CβS]).

Inter-individual variability (activity differences among subjects) was explained by the exponential relationships between the transformation rate constant from homocysteine to methionine (*K*_*HM*_, Eq. 8) and the distribution rate constant of homocysteine from the central to the peripheral compartment (*K*_*HP*_, Eq. 9). In addition, ZDF status was added to the MMC model as dichotomous covariate (0 or 1), to quantify diabetic effects on rate constants in the ZDF rats. Equations corresponding to the above explanations were as follows:

8 $$ {K}_{HM}={\theta}_{K_{HM}}\cdot {e}^{\left({\eta}_1+{\theta}_1\cdot ZDF\right)} $$

9 $$ {K}_{HP}={\theta}_{K_{HP}}\cdot {e}^{\left({\eta}_2+{\theta}_2\cdot ZDF\right)} $$

10 $$ {K}_{HC}={\theta}_{K_{HC}}\cdot {e}^{\left({\theta}_3\cdot ZDF\right)} $$

11 $$ {K}_{HC}={\theta}_{K_{HC}}\cdot {e}^{\left({\theta}_4\cdot ZDF\right)} $$

Residual variability was explained by a combined error model using both additive and proportional error (equation was not shown).

The performance of the MMC model was assessed using graphical and numerical diagnostic tools. The numerical analysis showed that the reduction in the objective function value (OFV) was at least 10.83 (*P* value < 0.001 for one degree of freedom). Diagnostic plots, including standard goodness-of-fit plots and graphical assessment of the distribution of conditional weighted residuals (CWRES), were also employed to establish whether the MMC model was adequate for describing the current dataset. In addition, the final model was evaluated via visual predictive check (VPC), where we graphically compared the original datasets and the simulated results, with 95% confidence intervals (CIs), at the 5th, 50th, and 95th percentiles.

### Use of the MMC model to simulate a methionine-rich diet

We assumed that methionine and homocysteine metabolisms were altered when T2DM patients ingested a methionine-rich diet for 60 days. To estimate the changes in two component levels after administration of a methionine-rich diet, 1000 virtual datasets were generated with the aid of Monte Carlo simulations. All simulated subjects were given 1 mmol/kg methionine divided into three times daily. For methionine and homocysteine concentrations, the simulated predicted accumulation ratio, total area under the curves (AUC) to total duration (AUC_total_), the AUC to 8 h (AUC_0-8h_), and the AUC to the last time point (AUC_last_) values, were compared between ZDF and control rats. The predicted accumulation ratio was the ratio of the AUC_8-16h_ to the AUC_0-8h_ (on day 1). The maximum concentration (*C*_*max*_), time to maximum concentration (*T*_*max*_), and AUC values were calculated via non-compartmental analysis (NCA).

### Statistical analysis

The MMC model building and simulation were performed using NONMEM® (ver. 7.3.0) assisted by Perl-Speaks-NONMEM (PsN, ver. 4.3.0). Model parameters were derived using first-order conditional estimation method with interactions permitted among the parameters (FOCE-INTER). NCA was conducted using Phoenix (ver. 6.4.0). For all tests, we considered a *P*-value < 0.05 to reflect statistical significance.

## Results

### A mathematical MMC model for ZDF rats

A new mathematical MMC model was successfully developed using plasma measurements of both methionine and homocysteine, and was characterized using seven compartments. From the prior study [14], we found that the plasma concentration kinetics of methionine were similar in the ZDF and control groups, while homocysteine exhibited a different kinetic pattern. Furthermore, since the hepatic concentrations of SAM and SAH did not differ between control and ZDF rats, the *K*_*CM*_, *K*_*MC*_, *K*_*MS*_, *K*_*SS*_, *K*_*EL*_, and *V*_*c*_ parameters were predicted without consideration of disease status. Changes in homocysteine levels in the ZDF rat, the left shift of *T*_*max*_, and the increment in *C*_*max*_, are all explained by an increment in *K*_*SH*_ and decrements in both *K*_*HM*_ and *K*_*HC*_. The *K*_*SH*_ value of ZDF rats was almost 16% greater than that of controls (ZDF versus control, 13.5 versus 11.6 h^− 1^). Although the homocysteine elimination slope was greater in ZDF rats in the early phase of elimination, such elimination was delayed after 210 h compared with that of controls, explained by decreases in both *K*_*HM*_ and *K*_*HC*_ at this time. The final model and kinetic parameters are shown in Table 1.

**Table 1:** Final estimated MMC model parameters

Parameters	Control rats		ZDF rats		IIV (%)
	Estimated value	RSE (%)	Estimated value	RSE (%)	
*K*_*CM*_ *(hr* ^*− 1*^ *)*	0.13	16.8	0.13	16.8	–
*K*_*MC*_ *(hr* ^*−1*^ *)*	0.0629	–	0.0629	–	–
*K*_*MS*_ *(hr* ^*−1*^ *)*	0.605	9.5	0.605	9.5	–
*K*_*SS*_ *(hr* ^*−1*^ *)*	3220	–	3220	–	–
*K*_*EL*_ *(hr* ^*−1*^ *)*	0.245	–	0.245	–	–
*Vc (L/kg)*	0.15	–	0.15	–	–
*K*_*SH*_ *(hr* ^*−1*^ *)*	11.6	20.5	13.5	106.6	–
*K*_*HM*_ *(hr* ^*−1*^ *)*	30.7	31.1	2.54	14.7	70.1
*K*_*HC*_ *(hr* ^*−1*^ *)*	11.1	4.8	0.52	668.9	–
*K*_*HP*_ *(hr* ^*−1*^ *)*	142	18.5	20.4	29.7	45.4
*K*_*PH*_ *(hr* ^*−1*^ *)*	5.13	10.4	0.032	16.0	–

*ZDF* zucker diabetic fatty, *IIV* inter-individual variability, *RSE* relative standard error, *K*_*CM*_ *& K*_*MC*_ inter-compartment rate constant between peripheral and methionine compartment, *K*_*MS*_ rate constant from methionine to *S*-adenosylmethionine, *K*_*SS*_ rate constant from *S*-adenosylmethionine to *S*-adenosyl homocysteine, *K*_*EL*_ elimination rate constant, V_c_ apparent volume of distribution of peripheral compartment, *K*_*SH*_ rate constant from *S*-adenosylhomocysteine to homocysteine, *K*_*HM*_ rate constant from homocysteine to methionine, *K*_*HC*_ rate constant from homocysteine to cysteine, *K*_*HP*_ & *K*_*PH*_ inter-compartment rate constant between homocysteine and peripheral compartment

**Fig. 2:**
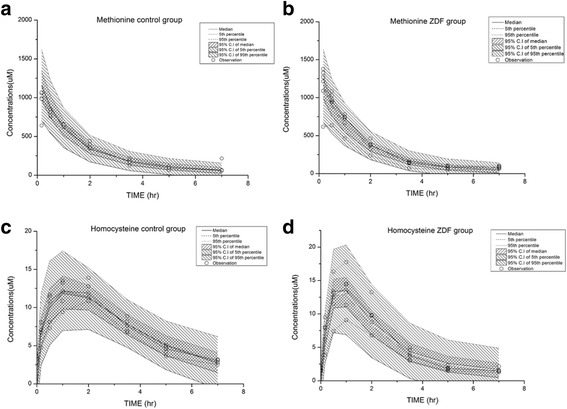
Visual predictive check (*n* = 1000) results of final Mechanistic Methionine Cycle (MMC) model: methionine changes in (**a**) control rats and (**b**) ZDF rats, and homocysteine changes in (**c**) control rats and (**d**) ZDF rats

The goodness-of-fit plot for the final model showed that the population predicted and individual predicted concentrations were in good agreement with the observed value. The CWRES versus time plot was unbiased (data not shown). The VPC plot successfully explained the plasma concentrations of methionine and homocysteine; the 95% CIs of the 5th, 50th, and 95th percentiles of the observational data were well-covered in the MMC model (Fig. 2).

### Simulation of a methionine-rich diet

**Fig. 3:**
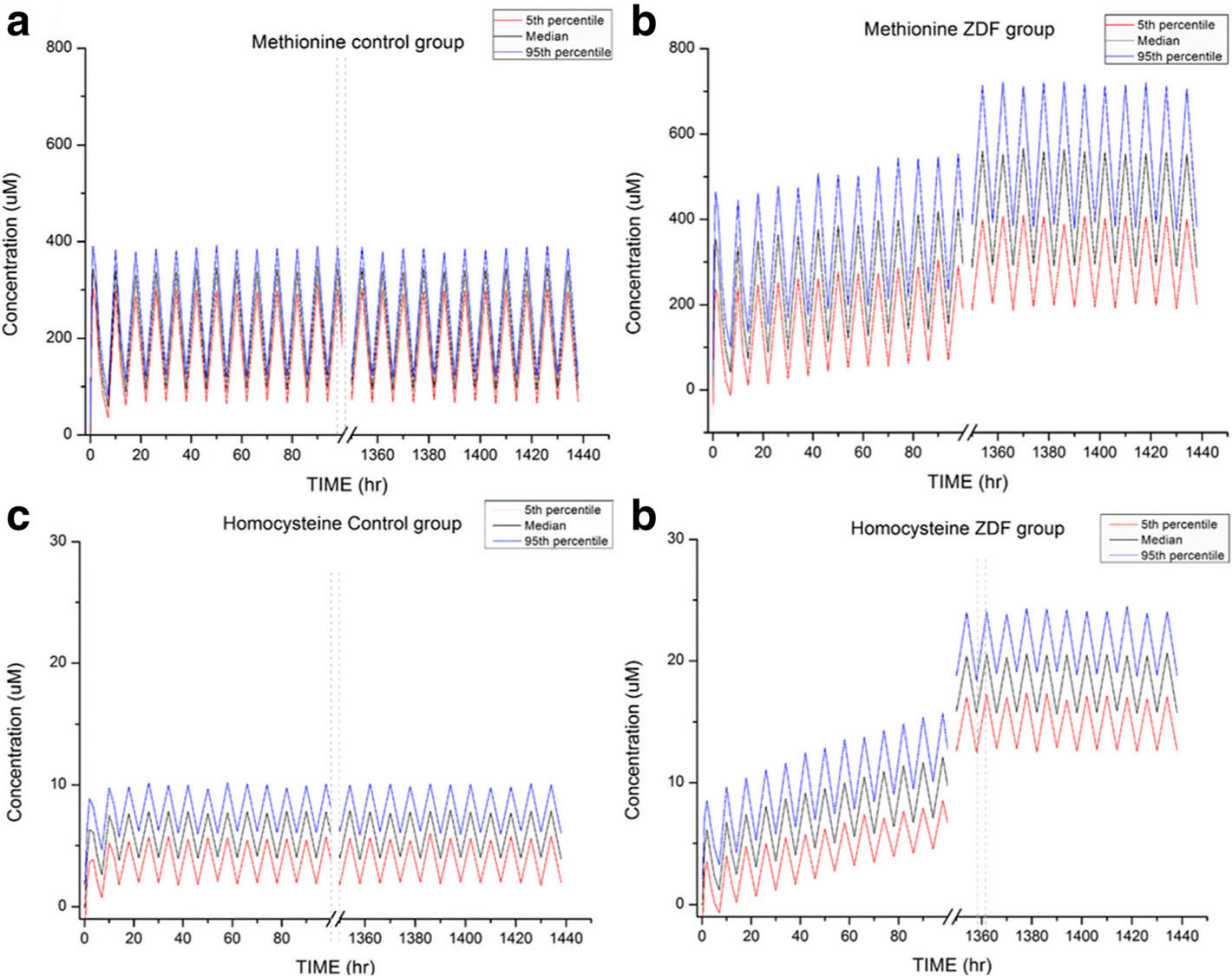
Plasma methionine levels of 1000 simulated subjects in (**a**) control rats and (**b**) ZDF rats, homocysteine levels of same subjects in (**c**) control rats and (**d**) ZDF rats

In the virtual scenario, both the methionine and homocysteine levels steadily increased in ZDF rats after high-dose methionine administration (Fig. 3). The concentrations of both components steadily increased over this time period and, after 60 days of administration, the level of methionine in the ZDF group was twice that of controls, triggering significant changes in the homocysteine level. Although the accumulation ratios of methionine and homocysteine at a dose of 0.8 mmol/kg methionine did not differ significantly between ZDF rats and controls (methionine in ZDF rats and controls, 1.22 and 1.10, *P* = 0.059; homocysteine in ZDF rats and controls, 1.23 and 2.78, *P* = 0.162), the ratio of homocysteine at a dose of 1 mmol/kg in ZDF rats was significantly greater than that in controls (ZDF rats and controls, 1.21 and 1.39, *P* = 0.003). At 1350 h, the AUC_8h_ of homocysteine was 307.84% greater in ZDF rats than controls (ZDF vs. control, 47.31 ± 6.06 vs. 145.64 ± 13.67; *P* < 0.001).

## Discussion

In the current study, we aimed to develop an MMC model using in vivo data under diabetic conditions, and to predict the changes in methionine and homocysteine levels after high-dose methionine administration. The final model successfully reproduced the kinetic profiles of methionine and homocysteine mathematically, and the elevated homocysteine levels were predicted for repeated high methionine ingestion. Thus, we confirmed the clinical importance of diet regulation in diabetic patients, for maintaining homocysteine levels within the normal range.

A prior study showed that *T*_*max*_ of homocysteine decreased and *C*_*max*_ increased in ZDF rats relative to normal rats, which may have resulted in homocysteine levels in the terminal phase being lower in ZDF rats than in the controls [14]. It seemed that the metabolism of homocysteine was upregulated under diabetic conditions [22]. More rapid transformation of methionine to homocysteine was related to rapid systemic distribution of homocysteine in patients of high-glucose status. The increased distribution of homocysteine during the initial phase was characterized by increased SAAH enzyme activity [23], as well as by higher protein levels in ZDF rats than in the controls [14]. The *K*_*SH*_ value of ZDF rats was higher than that of controls, which meant that upregulation of the insulin-signaling pathway induced the increased SAHH activity [23]. In addition, our model showed that elevated levels of enzymes catalyzing homocysteine production and recycling to methionine, corresponding with previous studies conducted using diabetic in vivo rat models [13, 24]. Among all catalyzing enzymes, BHMT played a significant role in catalyzing remethylation of homocysteine to methionine. In the early phase, BHMT activity was elevated under diabetic conditions, as a result of increased levels of mRNA encoding the enzyme. Thus, homocysteine was rapidly reduced, accompanied by remarkable reductions in the hepatic concentrations of betaine [24]. However, the catalyzing rate of homocysteine gradually decreased as betaine was depleted by the activation of BHMT in terminal phase [25]. Consequently, an accumulation of homocysteine occurred. Previously, we described how *K*_*HM*_ and *K*_*HC*_ value were lower in ZDF rats than in controls. On the basis of the calculated accumulation factor, we predicted that blood homocysteine levels would rise about three-fold after administration of methionine on three-times per day over 60 days (a total of 180 occasions). Furthermore, the decline in *K*_*HC*_ was associated with the decrease in the level of CβS, which is predominantly associated with the trans-sulfuration pathway [18]. A previous study revealed that hydrogen sulfide (H_2_S) signaling was impaired under diabetic conditions, which can contribute to mediating a reduction in CβS activity [26]. From these MMC modeling results, we confirmed that the metabolisms of homocysteine under diabetic conditions were different than those under normal conditions.

Subsequently, we explored the influence of a high-methionine diet on homocysteine levels in T2DM rats, on the basis of our model. The methionine dose was determined based on previous reports that have evaluated a high-dose methionine diet in rats. Yagisawa M et al. injected with methionine at a dose of 0.67 mmol/kg as a high-dose methionine diet in rats for estimating the influence of betaine on cardiovascular diseases. In the study of Cao Y et al., rats were treated with saline and intravenous high-dose methionine (0.8 mmol/kg) to generate hyperhomocysteinemia model. The methionine dose was equivalent to the human dose of 0.12 g/kg by scaling the body size, which was higher dose used in Haulrik N et al. study [27]. The data obtained after repeated methionine administrations over 2 months showed that homocysteine concentrations were higher in those with diabetes, regardless of individual differences in the enzymes of the MMC. The greater accumulation ratio of homocysteine with high-dose methionine led to a greater increase in the AUC of homocysteine, in the ZDF rats compared to the controls. This may imply an increased risk of hyperhomocysteinemia in diabetic patients. These results are consistent with those of previous studies that used protein-rich or high-fat diets. High amount of methionine intake induced more efficient homocysteine metabolism, explained by the adaptive activation of enzymes involved in the methionine cycle [27, 28]. Another study found that protein-rich breakfasts and dinners triggered acute diphasic changes in plasma methionine and homocysteine concentrations [29]. Thus, in diabetic patients, a high-methionine diet can induce significant variation in methionine metabolism, which is associated with an increased risk of vascular complications.

This mathematical approach is both useful and necessary when attempting to identify modes of regulation of complex metabolic systems and to predict changes in methionine and homocysteine profiles affected by various factors that cannot be investigated in the lab. Nevertheless, our study had certain limitations. First, the model did not evaluate clinical factors other than disease status. As the data were obtained in a previous well-controlled experiment using a rat model, clinical diversity was low and inter- and intra-subject variabilities could not be considered. In addition, the simulation results were analyzed by direct extrapolation from in vivo rat data to humans. However, it has been demonstrated that the several enzymes influencing plasma homocysteine profiles are similar in the rat and human [30].

## Conclusion

In conclusion, we found that methionine-rich foods elevated both methionine and homocysteine levels in ZDF model, regardless of individual differences in enzymes of the MMC cycle. It is well known that high homocysteine levels are significantly associated with both atherosclerosis and vascular disease, by inhibiting vascular endothelial cell growth [21], and hyperhomocysteinemia is considered to be a biomarker of atherosclerosis in mammals [31, 32], especially in patients with T2DM than those without [9]. Thus, it is important that T2DM patients avoid high-methionine diets to regulate their homocysteine levels within the normal range, and to prevent cardiovascular disease and mortality.

## Abbreviations

AUC: Area under the concentration curve
BHMT: Betaine-homocysteine methyltransferase
C_max_: Maximum concentration
CWRES: Conditional weighted residuals
CβS: Cystathionine beta-synthase
FOCE-INTER: First-order conditional estimation method with interactions
LC-ESI/MS/MS: Liquid chromatography-electrospray ionization/mass/mass
MMC: Methionine metabolism cycle
NCA: Non-compartmental analysis
NONMEM: Non-linear mixed effect modeling
OFV: Objective function value
SAH: *S*-adenosyl-*L*-homocysteine
SAHH: *S*-adenosyl-*L*-homocysteine hydrolase
SAM: *S*-adenosylmethionine
T2DM: Type 2 diabetic mellitus
T_max_: Time to maximum concentration
VPC: Visual predictive check
ZDF: Zucker Diabetic Fatty
